# Evolution of Microstructure and Residual Stress under Various Vibration Modes in 304 Stainless Steel Welds

**DOI:** 10.1155/2014/895790

**Published:** 2014-01-27

**Authors:** Chih-Chun Hsieh, Peng-Shuen Wang, Jia-Siang Wang, Weite Wu

**Affiliations:** Department of Materials Science and Engineering, National Chung Hsing University, 250 Kuo-Kuang Road, Taichung 402, Taiwan

## Abstract

Simultaneous vibration welding of 304 stainless steel was carried out with an eccentric circulating vibrator and a magnetic telescopic vibrator at subresonant (362 Hz and 59.3 Hz) and resonant (376 Hz and 60.9 Hz) frequencies. The experimental results indicate that the temperature gradient can be increased, accelerating nucleation and causing grain refinement during this process. During simultaneous vibration welding primary **δ**-ferrite can be refined and the morphologies of retained **δ**-ferrite become discontinuous so that **δ**-ferrite contents decrease. The smallest content of **δ**-ferrite (5.5%) occurred using the eccentric circulating vibrator. The diffraction intensities decreased and the FWHM widened with both vibration and no vibration. A residual stress can obviously be increased, producing an excellent effect on stress relief at a resonant frequency. The stress relief effect with an eccentric circulating vibrator was better than that obtained using a magnetic telescopic vibrator.

## 1. Introduction

Heat treatment is currently a primary method used for the relief of residual stress; its principle is to heat a sample to a suitable temperature and then hold it there for a set time. At high temperatures, the atoms are redistributed in metals and residual stress is relieved simultaneously. On the other hand, heat treatment has some limitations, for example, the formation of oxide film, deformation in materials, strength degradation, and the amount of time needed to apply the heat treatment. Hence, many new techniques have been developed to replace or improve these limitations [1, 2].

Simultaneous vibration welding is a new technique used to relieve residual stress in many steels. This technique can be used to decrease both residual stress and deformation as well as to increase the strength of materials. The benefits of this process include saving energy, no pollution, low cost, high efficiency, and ease of operation [3]. In general, the vibration modes can be divided according to the use of an eccentric circulating vibrator, a magnetic telescopic vibrator, a pneumatic vibrator, and a supersonic vibrator.

The aim of this study is to discuss the *δ*-ferrite content and residual stress in welds made using two vibration modes: an eccentric circulating vibrator and a magnetic telescopic vibrator. The mechanism of residual stress relief will also be investigated. The selected vibration frequencies were resonant (376 Hz, 60.9 Hz) and subresonant (362 Hz, 59.3 Hz) [4–8].

## 2. Experimental Procedures

### 2.1. Sample Preparation

AISI 304 stainless steel samples with dimensions 100 mm × 80 mm × 5 mm were selected. The real chemical composition of 304 stainless steel was analyzed using glow discharge spectroscopy (GDS) and is indicated in Table 1. The samples were cleaned with acetone (C_3_H_6_O) before welding in order to clean the oxide, dust, and grease off of them and they were then ground with SiC paper and polished with an Al_2_O_3_ powder paste.

### 2.2. The Simultaneous Vibration Welding System

**Figure 1:**
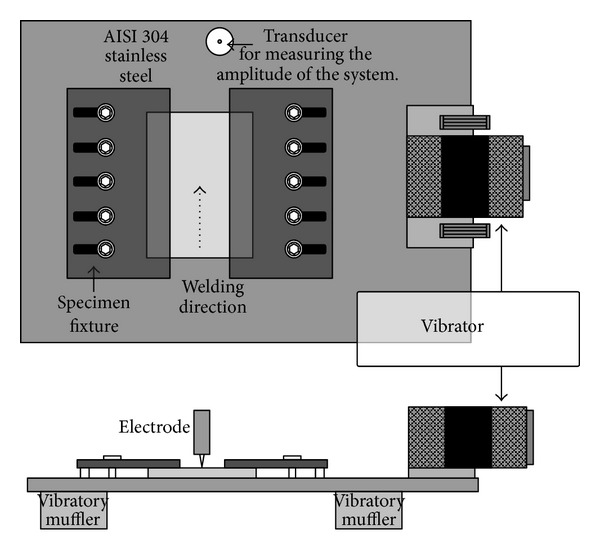
The simultaneous vibration welding system.

**Figure 2:**
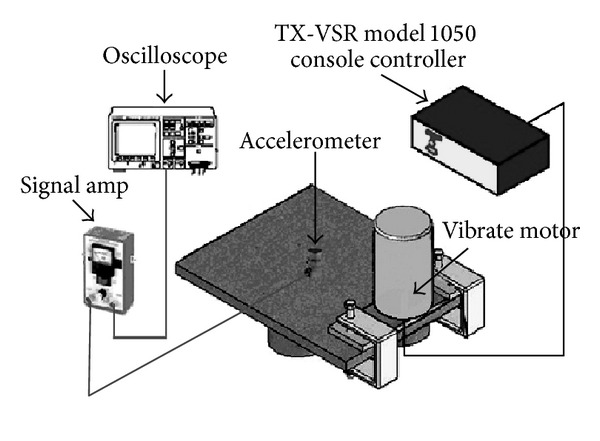
The oscilloscope examination system of the TX-VSR.

**Figure 3:**
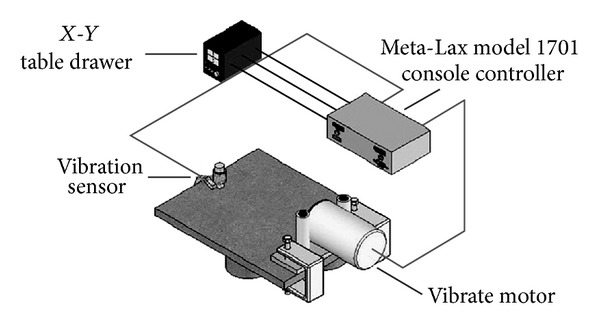
The oscilloscope examination system of the Meta-Lax.

**Figure 4:**
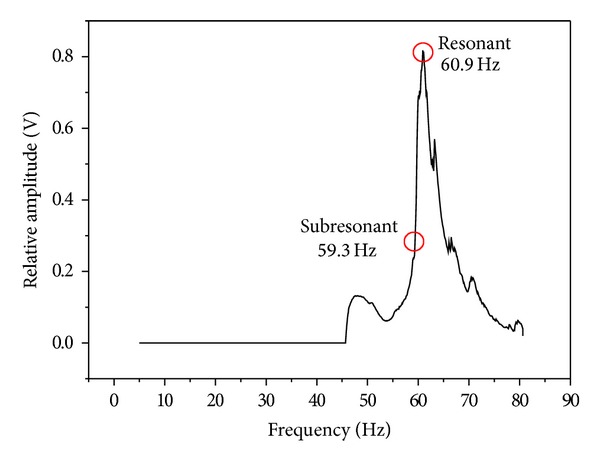
The spectrum map of the Meta-Lax.

The simultaneous vibration welding of 304 stainless steel was carried out without filler at an arc voltage of 10 V, a welding current of 120 A, and a travel speed of 120 mm/min. Detailed welding parameters are listed in Table 2. The vibrators in this study consisted of an eccentric circulating vibrator (Meta-Lax) and a magnetic telescopic motor (TX-VSR) set up at the welding working table, as shown in Figure 1. The vibration system for a magnetic telescopic motor was used to adjust the vibration controller from the lowest to the highest frequency in order to obtain a resonant frequency, as well as a display of the waveform on the oscilloscope using an accelerometer plus an amplifier. An examination of simultaneous vibration welding, as displayed on the oscilloscope, is shown in Figure 2. The vibration system for an eccentric circulating vibrator measured the amplitude of vibration of the working table using a vibration sensor, which transferred a signal to a Meta-Lax controller. From this process, the spectrum map was obtained from a table drawer, as shown in Figure 3. A spectrum map was constructed according to the *x*-axis (the opposite amplitude of vibration) and *y*-axis (vibration frequency), as shown in Figure 4. After the spectrum map was measured, the parameters of the vibration frequencies were selected as being resonant (TX-VSR: 60.9 Hz, Meta-Lax: 376 Hz) or subresonant (TX-VSR: 59.3 Hz, Meta-Lax: 362 Hz). Following this, the simultaneous vibration welding system was started.

### 2.3. Microstructural Observation

In order to observe the variation of *δ*-ferrite and *γ*-austenite in the 304 samples, aqua regia (HCl : HNO_3_ = 1 : 3) was used as an etchant. By using this, the *δ*-ferrite and *γ*-austenite were “attacked.” Observations concerning the morphologies of the *δ* and *γ* phases were carried out in the 304 samples using an optical microscope (ZEISS Axioskop 2 MAT, OM).

### 2.4. Measurement of the Retained *δ*-Ferrite

Cross sections of the 304 welded samples were prepared in order to measure the amount of retained *δ*-ferrite. Metallographic observation of the retained *δ*-ferrite in the 304 welded samples used a color metallographic technique to measure LB1 etchant (0.5 g K_2_S_2_O_5_ + 20 g NH_4_F·HF + 100 mL H_2_O) [9]. The retained *δ*-ferrite was observed with an optical microscope (ZEISS Axioskop 2 MAT, OM) and its phase fraction was examined using an image analyzer (Image-Pro Plus Ver. 5.0, IA) at various vibration frequencies.

### 2.5. X-Ray Diffraction Analysis

An X-ray diffractometer (MAC MXT-III, XRD), with Cu K*α* radiation, a scanning rate of 2°/min and a 2*θ* value from 40° to 100°, was utilized to analyze the 304 stainless steel after simultaneous vibration welding. The experimental operating voltage and current were 40 Kv and 30 mA, respectively.

### 2.6. Measurement of the Residual Stress

As is done with all diffraction methods, lattice spacing is calculated from the diffraction angle, 2*θ*, and the known X-ray wavelength determined using Bragg's Law. The precision necessary for strain measurement of engineering materials can be achieved using the diffraction peaks produced in the high back reflection region, where 2*θ* > 120 deg. The macrostrain is determined from shifts typically less than one degree in the mean diffraction peak position.

The strain in the sample surface at an angle *φ* from the principal stress *σ*_11_ is then given by

(1) $$\begin{array}{c} \varepsilon_{\varphi \psi }=\left(\frac{1+\nu }{E}\right)\sigma_{\psi }\sin^{2}\psi -\left(\frac{\nu }{E}\right)\left(\sigma_{11}+\sigma_{22}\right), \end{array}$$

where *ε* is the strain, *ν* is the Poisson ratio, *E* is Young's modules, and *σ* is the stress (MPa).

If *d*_*φψ*_ is the spacing between the lattice planes measured in the direction defined by *φ* and *ψ*, the strain can be expressed in terms of the changes in spacing within the crystal lattice:

(2) $$\begin{array}{c} \varepsilon_{\varphi \Psi }=\frac{d_{\phi ,\psi }-d_{0}}{d_{0}}, \end{array}$$

where *d*_0_ is the stress-free lattice spacing. Substituting into (1) and solving for *d*_*φψ*_ yield

(3) dφΨ=[(1+νE)(hkl)σψd0]sin2ψ −(νE)(hkl)d0(σ11+σ22)+d0.

The lattice spacing *d*_*φψ*_ is a linear function of sin^2^ *ψ*:

(4) $$\begin{array}{c} \left(\frac{\partial d_{\phi \psi }}{\partial \text{si}{\text{n}}^{2}\Psi }\right)={\left(\frac{1+\nu }{E}\right)}_{\left(\text{hkl}\right)}\sigma_{\psi }d_{0},\\ \sigma_{\psi }={\left(\frac{E}{1+\nu }\right)}_{\left(\text{hkl}\right)}\frac{1}{d_{0}}\left(\frac{\partial d_{\phi \psi }}{\partial \text{si}{\text{n}}^{2}\Psi }\right). \end{array}$$

An X-ray diffractometer (MAC MXT-III, XRD) was used for the measurement of residual stress. The outside weld bead area was covered by a thin copper plate to prevent contamination by diffraction signals.

### 2.7. Measurement of Vickers Hardness

The hardness value of welds was measured by a Micro-Hardness tester (Shimadzu HMV-2, Vickers Hardness Tester) with a 0.98 N load applied for 10 sec at a quarter plane from the specimen surface of the cross section. The measured position was at the center of the welds with the measured distance between the two points being 0.5 mm. This test determined the variation in the hardness of the welds under different vibration frequencies.

## 3. Results and Discussions

### 3.1. Spectrum Analysis

**Figure 5:**
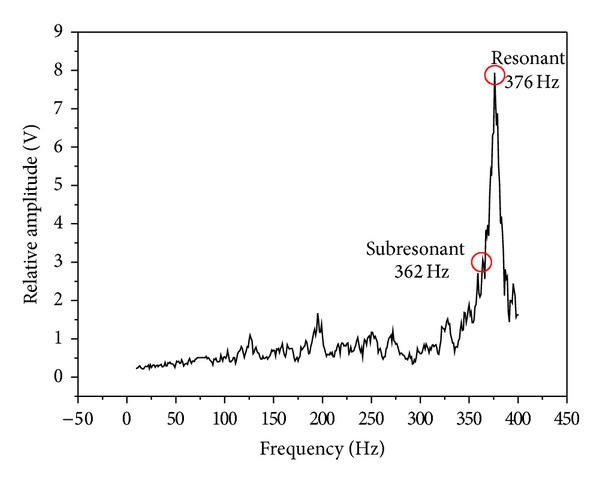
The spectrum map of the TX-VSR.

**Figure 6:**
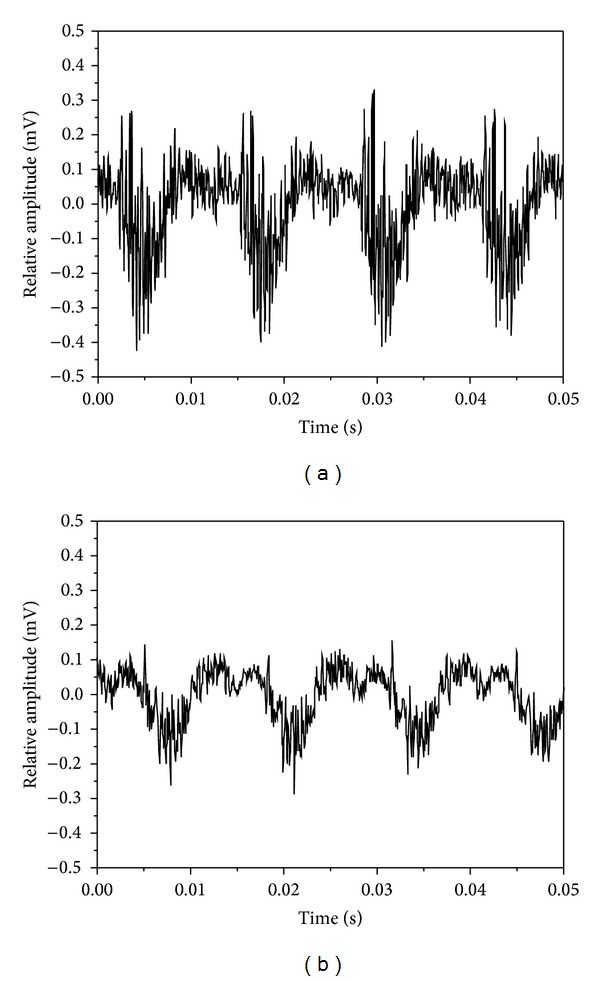
The waveform of the Meta-Lax (a) resonant: 60.9 Hz and (b) subresonant: 59.3 Hz.

**Figure 7:**
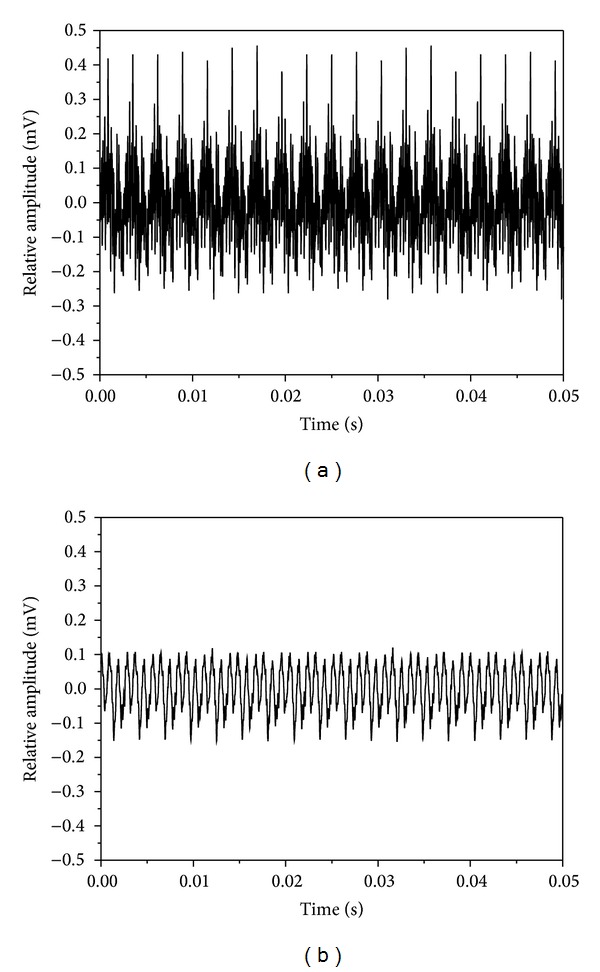
The waveform of the TX-VSR (a) resonant: 375 Hz and (b) subresonant: 362 Hz.

Figure 4 shows the spectrum map created using an eccentric circulating vibrator. It indicates maximum amplitude of vibration at a resonant frequency of 60.9 Hz. According to Meta-Lax technology [10], the subresonant point is within a former 10 Hz of the maximum amplitude of vibration and resonant frequency of 1/3. In this study, the former 10 Hz of resonance decayed rapidly on the spectrum map so the amplitude of vibration of 1/3 (59.3 Hz) was selected as a subresonant point.  Figure 5 shows the spectrum map created using a magnetic telescopic vibrator with the resonant point occurring at a frequency of 375 Hz. Here, the resonant frequency of 1/3 was selected as 362 Hz. In comparing the two spectrum maps, the resonant and the subresonant frequencies for a magnetic telescopic vibrator were about 6 times greater than those created using an eccentric circulating vibrator.  Figure 6 shows the waveform of the resonant and subresonant frequencies of an eccentric circulating vibrator. In this instance, the vibrator was set on the working table and the table and samples were vibrated by an eccentric circulating vibrator. As a result, the measured waveform was affected by the loss of energy transfer and the energy absorption of the samples, meaning that the waveform was not the sine wave.  Figure 7 indicates the waveform of the resonant and subresonant frequencies of a magnetic telescopic vibrator. In this experiment, this waveform is indicated as a continuous knock wave. The peck voltage of the opposite amplitude of vibration (*V*_pp_ = ±0.42 mV) is the same on the waveform of the resonant frequencies for the two vibration systems. However, the peck voltage of the opposite amplitude of vibration at a subresonant frequency is about equal to the opposite amplitude of vibration of the resonant frequency of 1/3 (±0.14 mV~0.16 mV). On the other hand, a vibration waveform of high frequency occurs near the primary wave of an eccentric circulating vibrator in comparison to four vibration waveforms. It means that the samples sustained a microvibration in addition to the original vibration. The resonant waveform of the magnetic telescopic vibrator displayed the same result. Liao [11] pointed out that the microvibration of a high frequency wave can cause a great amount of microdeformation and has a greater effect on residual stress relief. However, the high frequency wave of the subresonant frequency is not obvious in a magnetic telescopic vibrator. All output energies were transferred into vibration energies and that the plastic deformation of samples was unapparent. The effect of the residual stress relief was therefore limited.

### 3.2. Microstructural Analysis

**Figure 8:**
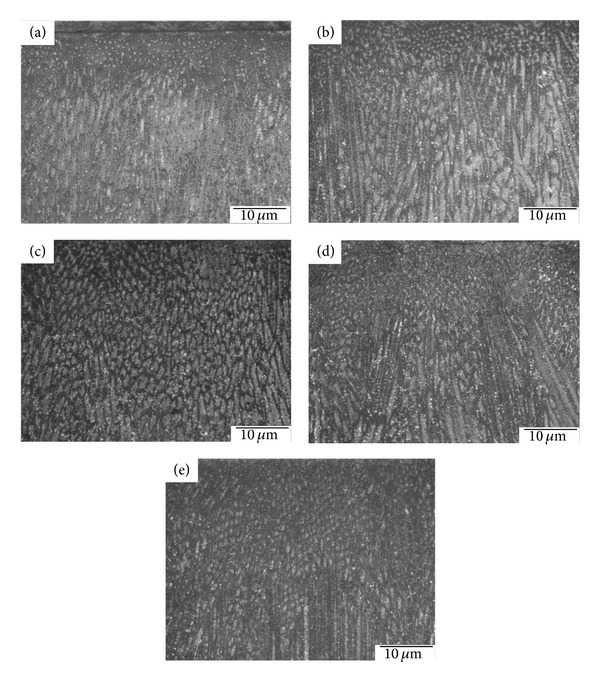
The microstructures in the center of the welds with various vibration frequencies: (a) 0 Hz, (b) TX-VSR 362 Hz, (c) TX-VSR 375 Hz, (d) Meta-Lax 59.3 Hz, and (e) Meta-Lax 60.9 Hz.

Figures 8(a)–8(e) show the weld microstructures of 304 stainless steel with and without vibration. As shown in Figure 8(a), fine dendrites grew at the top of the welds without vibration and dispersed columnar grains were observed. Dendrites were found in the bottom of the welds for the subresonant and resonant frequencies produced by the magnetic telescopic vibrator, as shown in Figures 8(b)-8(c). Concerning solidification in the bottom of welds, vibration welding made it difficult to retard dendritic growth because of a higher degree of supercooling. The center of the welds indicated many equiaxed grains created at a subresonant frequency and, in addition, exhibited many dendritic grains at a resonant frequency. Figures 8(d)-8(e) show the weld microstructures created at subresonant and resonant frequencies using an eccentric circulating vibrator; similar grain refinement was displayed at a resonant frequency. However, perturbation motion can occur from the molten pool during vibration and the dendrites can be broken at the same time. Nucleation sites will be increased when broken dendrites are formed. On the other hand, the distribution of temperature is uniform during vibration welding, meaning that the temperature difference is small [12]; therefore, the effect of vibration should be the temperature gradient (*G*). Wei [4] pointed out that the vibration can accelerate the flow of liquid metal and redistribute the temperature of the weld pool. Hence, the temperature gradient can be decreased during solidification, and then the equiaxed grains are formed at the same time.

**Figure 9:**
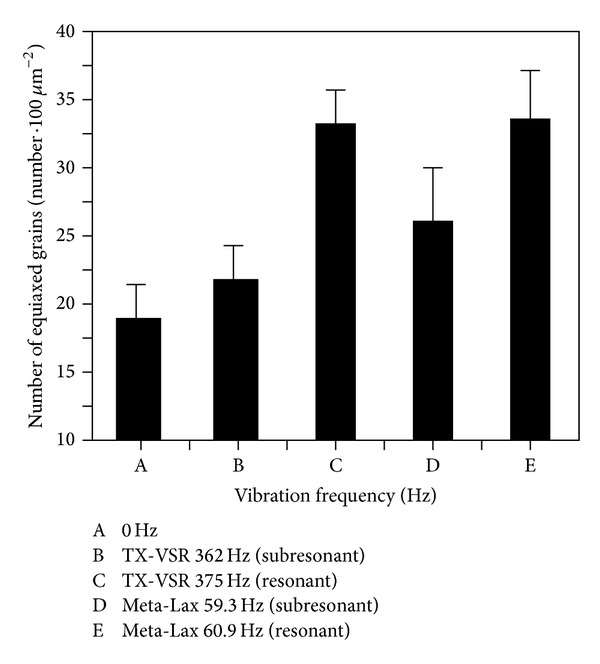
The amount of the equiaxed grain in the welds.

On the other hand, vibration can increase the contact possibility at the solid-liquid (S-L) interface and help the supercooling of the liquid to nucleate. Consequently, the nucleation point can be raised with lots of the fine equiaxed grains being formed [13].  Figure 9 shows the number of equiaxed grains per unit area (100 *μ*m^2^). High quality equiaxed grains mean that the nucleation point is greater, so the grain size becomes smaller. This result indicates that vibration can cause grain refinement of the primary *δ*-ferrite. But the difference in grain refinement was not obvious in the two vibration modes. The retardation of dendrites using a magnetic telescopic vibrator is better than the level achieved using an eccentric circulating vibrator.

### 3.3. X-Ray Diffraction Analysis

**Figure 10:**
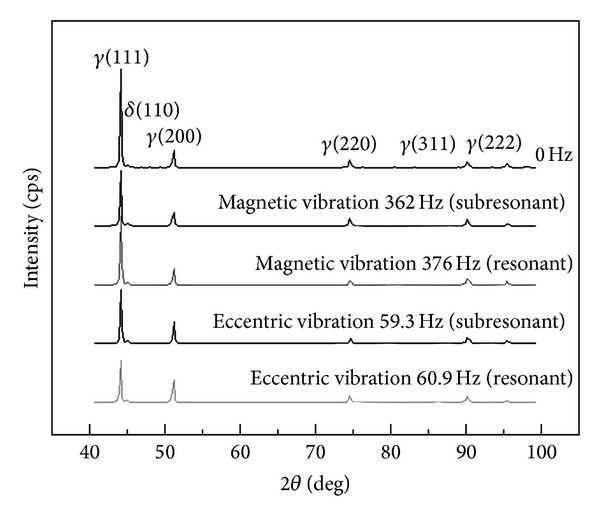
X-ray diffraction pattern of the welds with different vibration frequencies.

Figure 10 indicates the diffraction pattern of various vibration modes at resonant and subresonant frequencies. The results indicate that the primary phase included the *δ*-ferrite and the *γ*-austenite in the 304 stainless steel. The diffracted peak of the *γ*(111) had the highest intensity without vibration and with vibration. The intensity ratio of *γ*(111) and *γ*(200) was about 5 : 1 without vibration. However, the intensity ratio of the *γ*(111) and *γ*(200) was 2.5 : 1 at a resonant frequency using an eccentric circulating vibrator.  Table 3 shows the examined results of full width at half maximum (FWHM). The width of the FWHM in different crystallographic planes of *γ*(111), *γ*(200), *γ*(220), and *δ*(110) increases after vibration. Kuo [13] has pointed out that the increment of the FWHM is due to the stacking fault after the simultaneous vibration welding. Hence, the mechanism of stress relief for vibration welding is attributed to formation of the stacking fault.

Observing the diffracted peak ratio, the intensity of the *γ*(200) does not exceed that of the *γ*(111) after vibration, but the diffracted peak ratio of the *γ*(111) decreased vibration. Kuo [13] reported that vibration welding can change the crystallization direction and strengthen the preferred orientation. Because the *δ* → *γ* phase transformation occurs after the vibration welding of 304 stainless steel, the preferred orientation will be changed. For an FCC structure with closest packing of the *γ*(111), the diffracted peak ratio was lower with vibration than it was without vibration. This means that the crystallization ability of the *γ*-austenite in the *γ*(111) plane was low.

### 3.4. The Measurement of the *δ*-Ferrite Content

**Figure 11:**
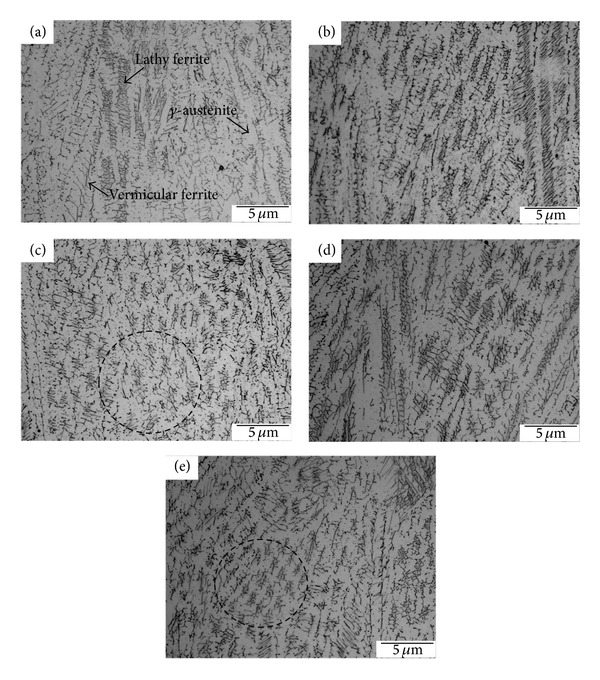
The morphologies of the *δ*-ferrite with two vibration modes: (a) 0 Hz (b) TX-VSR 362 Hz, (c) TX-VSR 375 Hz, (d) Meta-Lax 59.3 Hz, and (e) Meta-Lax 60.9 Hz.

**Figure 12:**
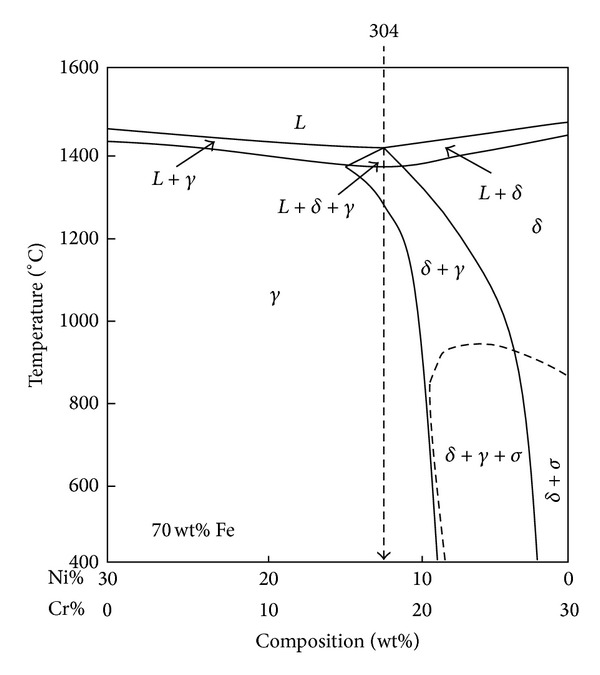
The Fe-Cr-Ni phase diagram.

Figures 11(a)–11(e) show the morphologies of *δ*-ferrite with various vibration modes. The parts of dark and light were the *δ*-ferrite and *γ*-austenite, respectively. The *δ*-ferrite indicated two morphologies of the lathy and the vermicular. However, the solidification path is as follows: *L* → (*L* + *δ*)→(*L* + *δ* + *γ*)→(*δ* + *γ*)→(*γ*) in the AISI 304 stainless steels. The vibration welding can affect the diffusion of liquid atoms, but it can also affect the *δ* → *γ* phase transformation subsequent cooling. Because the *δ* → *γ* phase transformation occurs above 1400°C, as shown in Figure 12 [14]. Hence, the *δ* → *γ* solid phase transformation will occur during solidification. The *δ*-ferrite was a primary phase and the *δ* → *γ* phase transformation occurs because of the diffusion of Cr and Ni [15]. Therefore, the morphology of the residual *δ*-ferrite was affected by that of the primary *δ*-ferrite.

Figure 11(a) shows the morphology of the residual *δ*-ferrite in the welds without vibration, and the residual *δ*-ferrite indicates a continuous precipitation. The primary *δ*-ferrite is a columnar grain so that the residual *δ*-ferrite formed a continuous morphology of the primary *δ*-ferrite. Similar results can also be observed in Figures 11(b) and 11(d). As previously described in Section 3.2, the equiaxed grains were not enlarged at a subresonant frequency and many dendrites were formed in the welds; however, the residual *δ*-ferrite became discontinuous morphologies in the some local regions, as shown in Figures 11(c) and 11(e). Furthermore, the morphology of the primary *δ*-ferrite can be refined as the equiaxed grain at a resonant frequency; consequently, the morphology of the residual *δ*-ferrite is discontinuous at a resonant frequency.

**Figure 13:**
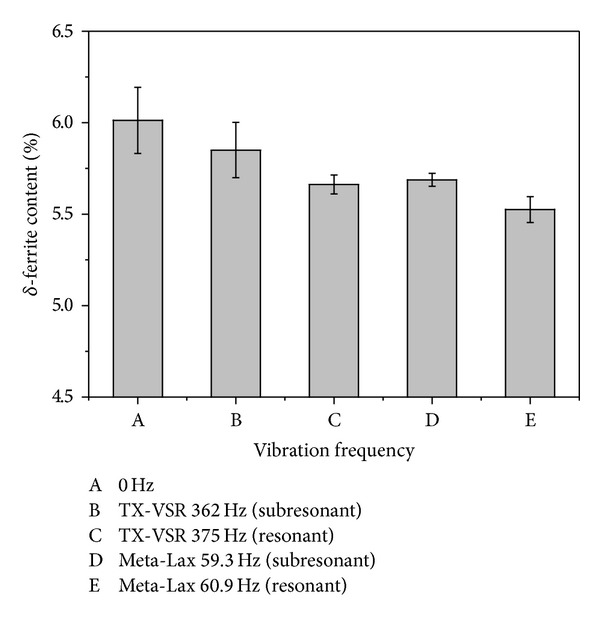
The *δ*-ferrite content of two vibration modes.

Figure 13 shows the residual *δ*-ferrite content using a Ferritescope. The residual *δ*-ferrite decreased after vibration welding. The decrement of the residual *δ*-ferrite is more obvious using an eccentric circulating vibrator than it is when using a magnetic telescopic vibrator. The atomic mobility of Cr and Ni in the weld pool of the 304 stainless steel can be accelerated during vibration. When Ni is easy to diffuse within the austenitic matrix, the *δ*-ferrite phase will be decreased. On the other hand, the *δ*-ferrite can be refined with vibration welding with a reduction in residual *δ*-ferrite occurring at the same time.

### 3.5. Residual Stress Analysis

**Figure 14:**
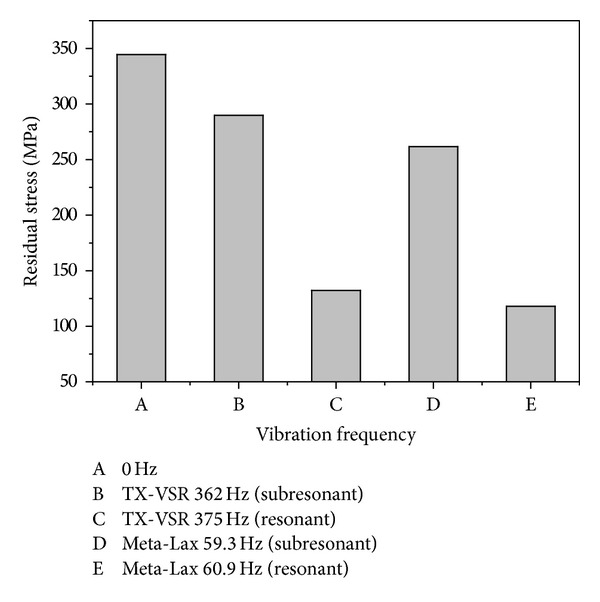
The relationship between the vibration frequency and the residual stress.

Figure 14 shows the relationship between residual stress and the vibration mode. The residual stress is about 344 MPa without vibration and it was decreased at subresonant (289 MPa) and resonant (132 MPa) frequencies using a magnetic telescopic vibrator (TX-VSR). However, the residual stress was also decreased at subresonant (261 MPa) and resonant (117 MPa) frequencies using an eccentric circulating vibrator (Meta-Lax). The above results indicate that residual stress can be relieved after vibration welding; the effect of the stress relief is excellent at resonant frequency. The decreased level of the TX-VSR and the Meta-Lax at a resonant and subresonant frequency reached 61% and 66% and 14% and 24%, respectively. The experimental results indicated that high vibration frequency had no beneficial effect on residual stress relief. There appears to be no direct proportional relationship between vibration frequency and residual stress.

### 3.6. Hardness Analysis

**Figure 15:**
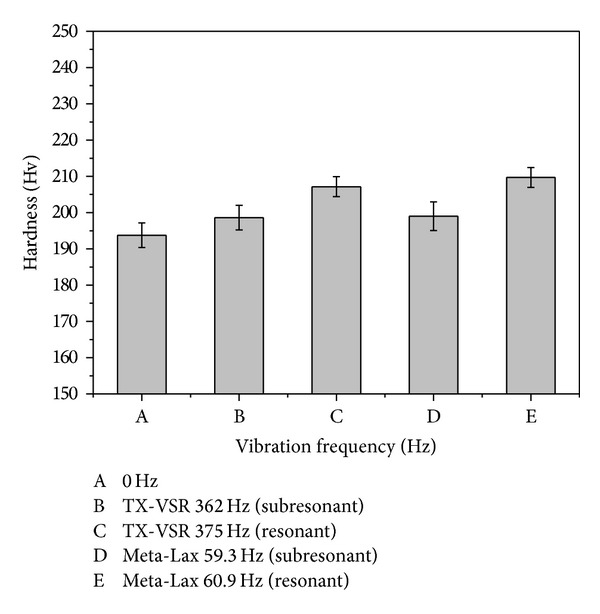
The relationship between the vibration frequency and the hardness value.

Figure 15 shows the relationship between vibration frequency and hardness after vibration welding. The hardness value without vibration was 193.8 Hv. Hardness values at subresonant and resonant frequencies using a magnetic telescopic vibrator (TX-VSR) were 198.6 Hv and 207.2 Hv, respectively. The hardness value at subresonant and resonant frequencies using eccentric circulating vibrator (Meta-Lax) were 199 Hv and 209.7 Hv, respectively. The above results indicate that the hardness had its lowest value without vibration and that the highest values were achieved at a resonant frequency in all conditions. The hardness value can be increased at a subresonant and a resonant frequency by about 2.5% and 7.3%, respectively. The increment of the hardness is due to grain refinement during vibration welding. Lu et al. [9] reported that vibration welding can raise the nucleation sites, leading to grain refinement and increasing the mechanical properties at the same time. On the other hand, the hardness values increase with the decrease of *δ*-ferrite content (as in Section 3.4) when the vibration frequencies are raised because the *δ*-ferrite is softer than *γ*-phase.

## 4. Conclusions

In this study, the effect of vibration on the microstructure and the residual stress in 304 stainless steel during welding were investigated. Two vibration modes were selected: an eccentric circulating vibrator (Meta-Lax) and a magnetic telescopic vibrator (TX-VSR). The significant results can be summarized as follows.

1. Equiaxed grains can be refined, retarding the formation of columnar grains in the welds at resonant frequencies using the Meta-Lax and TX-VSR.
2. The reduction of residual *δ*-ferrite using the Meta-Lax is more obvious than is the case in using the TX-VSR.
3. The relief of residual stress was excellent at a resonant frequency with two vibration modes: the Meta-Lax and the TX-VSR.
4. The hardness value of welds can be increased by up to 7.3% after vibration welding due to grain refinement.

## Figures and Tables

**Table 1:** Chemical composition of the AISI 304 stainless steel.

Element	Cr	Ni	Mn	Mo	Co	Cu	Si	C	Fe
wt.%	17.28	8.65	0.86	0.12	0.12	0.36	0.48	0.05	Bal.

**Table 2:** The welding parameters of the simultaneous vibration welding.

Welding process	GTAW
Arc current	120 A
Arc voltage	10 V
Traveling speed	120 mm/min
Protective gas	Argon, 15 L/min
Electrode	Tungsten, *φ*: 3.2 mm
Preheat	None
Filler	None

**Table 3:** XRD data of different crystallographic planes after vibration welding.

Vibration mode	2*θ*	Intensity	FWHM
*γ*(111)			
0 Hz	43.64	4378	0.168
TX-VSR (362 Hz)	43.61	2701	0.176
TX-VSR (375 Hz)	43.66	3372	0.178
Meta-Lax (59.3 Hz)	43.67	2823	0.174
Meta-Lax (60.9 Hz)	43.67	2804	0.182

*γ*(200)			
0 Hz	50.77	867	0.212
TX-VSR (362 Hz)	50.75	931	0.223
TX-VSR (375 Hz)	50.80	819	0.233
Meta-Lax (59.3 Hz)	50.81	1035	0.218
Meta-Lax (60.9 Hz)	50.81	1042	0.239

*γ*(220)			
0 Hz	74.71	397	0.288
TX-VSR (362 Hz)	74.70	350	0.301
TX-VSR (375 Hz)	74.74	261	0.303
Meta-Lax (59.3 Hz)	74.76	320	0.298
Meta-Lax (60.9 Hz)	74.76	322	0.307

*δ*(110)			
0 Hz	44.57	100	0.170
TX-VSR (362 Hz)	44.54	132	0.203
TX-VSR (375 Hz)	44.52	192	0.210
Meta-Lax (59.3 Hz)	44.60	128	0.185
Meta-Lax (60.9 Hz)	44.50	118	0.197
